# Dimensional engineering of a topological insulating phase in Half-Heusler LiMgAs

**DOI:** 10.1038/s41598-021-85806-1

**Published:** 2021-03-19

**Authors:** Raghottam M. Sattigeri, Prafulla K. Jha

**Affiliations:** Department of Physics, Faculty of Science, The Maharaja Sayajirao University of Baroda, Vadodara, Gujarat 390002 India

**Keywords:** Topological insulators, Electronic properties and materials, Spintronics

## Abstract

We propose a novel technique of dimensional engineering to realize low dimensional topological insulator from a trivial three dimensional parent. This is achieved by confining the bulk system to one dimension along a particular crystal direction, thus enhancing the quantum confinement effects in the system. We investigate this mechanism in the Half-Heusler compound LiMgAs with face-centered cubic (FCC) structure. At ambient conditions the bulk FCC structure exhibits a semi-conducting nature. But, under the influence of high volume expansive pressure (VEP) the system undergoes a topological phase transition (TPT) from semi-conducting to semi-metallic forming a Dirac cone. At a critical VEP we observe that, spin-orbit coupling (SOC) effects introduce a gap of $\sim$ 1.5 meV in the Dirac cone at high symmetry point $\Gamma$ in the Brillouin zone. This phase of bulk LiMgAs exhibits a trivial nature characterized by the ${\mathbb {Z}}_2$ invariants as (0,000). By further performing dimensional engineering, we cleave [111] plane from the bulk FCC structure and confine the system in one dimension. This low-dimensional phase of LiMgAs has structure similar to the two dimensional ${\text {1T-MoS}}_2$ system. Under a relatively lower compressive strain, the low-dimensional system undergoes a TPT and exhibits a non-trivial topological nature characterized by the SOC gap of $\sim$ 55 meV and ${\mathbb {Z}}_2$ invariant $\nu$ = 1. Although both, the low-dimensional and bulk phase exhibit edge and surface states, the low-dimensional phase is far more superior and exceptional as compared to the bulk parent in terms of the velocity of Fermions (${\text {v}}_f$) across the surface states. Such a system has promising applications in nano-electronics.

## Introduction

Over the past two decades Topological Insulators (TI) have sparked excitement in the scientific community for their non-trivial behaviour of insulating bulk and conducting surfaces^1^. In bulk (three dimensional) systems, it began with the study of quintuple layered systems like ${\text {Bi}}_{(1-x)} {\text {Sb}}_x$^2^, ${\text {Bi}}_{2} {\text {Te}}_{3}$, ${\text {Bi}}_{2} {\text {Se}}_{3}$, ${\text {Sb}}_{2} {\text {Te}}_{3}$^3^ which were found to be TI by the virtue of strong spin-orbit coupling (SOC) due to the constituent heavy (high atomic number) elements. These studies were an extension of the analogous quantum spin hall effect in low dimensional materials which was initially predicted in graphene^4^ and later on observed in quantum wells of HgTe-CdTe^5,6^. Since then, several materials in bulk as well as low dimensional phases have been predicted as TI^7,8^. As an extension to these predictions, other than the conventional SOC, several studies indicate towards methods by which TI nature can be realized in materials under the influence of Topological Phase Transition (TPT). For example, (i) strain/pressure induced band engineering^9–11^, (ii) variation of concentration (x) of constituent elements^2^ and variation of thickness (d)^5^ or electrically controlled number of layers (n) of the atoms in material^12^ and (iii) van der Waals layered systems or stacking effect^13,14^. Such methods alter the orbital nature or enhance the SOC driving the system through a TPT. Off the materials predicted so far, some are also known for their multi-functional properties other than TI nature like, ${\text {Bi}}_{2} {\text {Te}}_{3}$ and ${\text {Bi}}_{2} {\text {Se}}_{3}$ have been extensively investigated for their thermo-electric properties^15^. Half-Heusler’s (HH) are one such family of compounds which are known for their multi-functional properties^16–23^.

The materials from HH family based on Lithium (Li) have been extensively studied for their multi-functional properties such as, semi-conducting^24^, piezo-electric^21^, thermo-electric^22^ and TI^25,26^. But, there is a lack of study pertaining to the strain driven TI nature in Li based HH compounds. We began our investigation for TI nature in such HH systems of the form LiMgX (where X is element from Pnictogen family; As, Sb, Bi). Intrinsically, these compounds (LiMgBi and LiMgSb) exhibit semi-conducting^21,27,28^ nature which can be driven into TI nature by TPT driven by pressure^27,28^. We found that, LiMgBi is a strong TI^27^ as compared to LiMgSb which is a trivial insulator under the influence of pressure^28^. This was expected since, Bi is a heavier element as compared to Sb. It was speculated that, it may be possible to realize a strong TI nature in compounds (like LiMgSb) by confining the system in one dimension forming quantum well like HgTe-CdTe^29^. As far as the low dimensional phase is concerned, several elemental monolayers have been predicted post graphene^30^, such as, silicene^31,32^, germanene^32^, stanene^32^, arsenene^34^ and antimonene^35^. These monolayers with buckled hexagonal structure exhibit TI nature. Essentially, such systems were predicted to be TI by the virtue of their higher atomic number which results in stronger SOC. It was also found that, buckling is another parameter which affects the strength of SOC^33^. But unconventionally, it was predicted that, TI nature can also be realized under the influence of a tensile strain as in the case of arsenene and antimonene^34,35^. Hence, in the current study we use Density Functional Theory (DFT) based *first-principles* calculations to investigate the TI nature driven by pressure/strain in bulk phase of HH LiMgAs and its engineered low dimensional phase.

The bulk phase of LiMgAs has a *face-centred cubic* (FCC) structure^21^ of F${\overline{4}}$3m[216] space group. In its bulk phase, we observe odd number of band inversion in the Brillouin Zone (BZ) under Volume Expansive Pressure (VEP)^27,28^ which is discussed in terms of exchange in the orbital contributions near the Fermi level. This phase transition occurs at a high pressure of the order of 8.18 GPa, which can be useful for ultra-high pressure applications. But, the bulk phase of LiMgAs undergoes a TPT which exhibits a trivial nature. Since LiMgAs is a HH compound, it has zinc blende type sub-lattice^36^ formed by Mg-As, this results in band orders near the Fermi level similar to that of HgTe. Such band order arises in HH due to their adiabatic connection of the band topologies with HgTe type system^36^. Here, the band order are represented by ${\Gamma _8}$ for p-type orbital and ${\Gamma _6}$ for s-type orbital. We then quantify the TPT in terms of the band inversion strength defined as, $\Delta = {\text {E}}_{\Gamma _8} - {\text {E}}_{\Gamma _6}$^36,37^.

In order to achieve a TI nature at a relatively lower pressure, we reduce the dimensions of the system by confining it into one dimension. Such studies are known to unravel exciting properties of the materials as in the case of NbN, where the [100] and [111] crystal planes of the parent rocksalt structure exhibit strong Photocatalytic and Piezoelectric properties^38^. This method gives us two folded advantage, (a) reduction in area reduces the amount of pressure required for TPT and (b) the quantum size/confinement effects govern the orbital nature and SOC similar to HgTe-CdTe system. Here, we achieve this by confining the HH system in one dimension along the [111] crystal direction which has two phases, *hexagonal* (${\text {P6}}_3$/mmc[194] space group) and *trigonal* (P${\overline{3}}$m1[164] space group) similar to ${\text {2H}}_c$-${\text {MoS}}_2$ and ${\text {1T-MoS}}_2$ structures^39^ respectively. We find that, the quantum confinement of the system gives rise to a TI nature at a relatively lower strain of the order of $\sim$ 3 GPa as compared to the bulk parent. This technique is analogous to the variation of thickness of the material (which is proved to be one of the useful techniques to realize TI nature in low dimensional materials). The bulk as well as low dimensional phase of LiMgAs exhibit a dissipationless Dirac dispersion along the surface/edge states respectively. We qualitatively quantify the strength of the TI nature in terms of band inversion strength^36,37^ which is found to be higher in low dimensional phase as compared to the bulk phase. We further classify the system into topological class by computing the ${\mathbb {Z}}_2$ invariant^40^ for the bulk (($\nu _0, \nu _1\nu _2\nu _3$) $\simeq$ (0,000)) as well as low dimensional ($\nu$ = 1) phase around the Wannier Charge Centres^41,42^. With this study we propose low dimensional [111] layer of HH LiMgAs as a novel material based on dimensional engineering for; perspective applications in spintronic and nano-electronic devices^43^.

## Results and discussion

### Structural and dynamic properties

**Figure 1:**
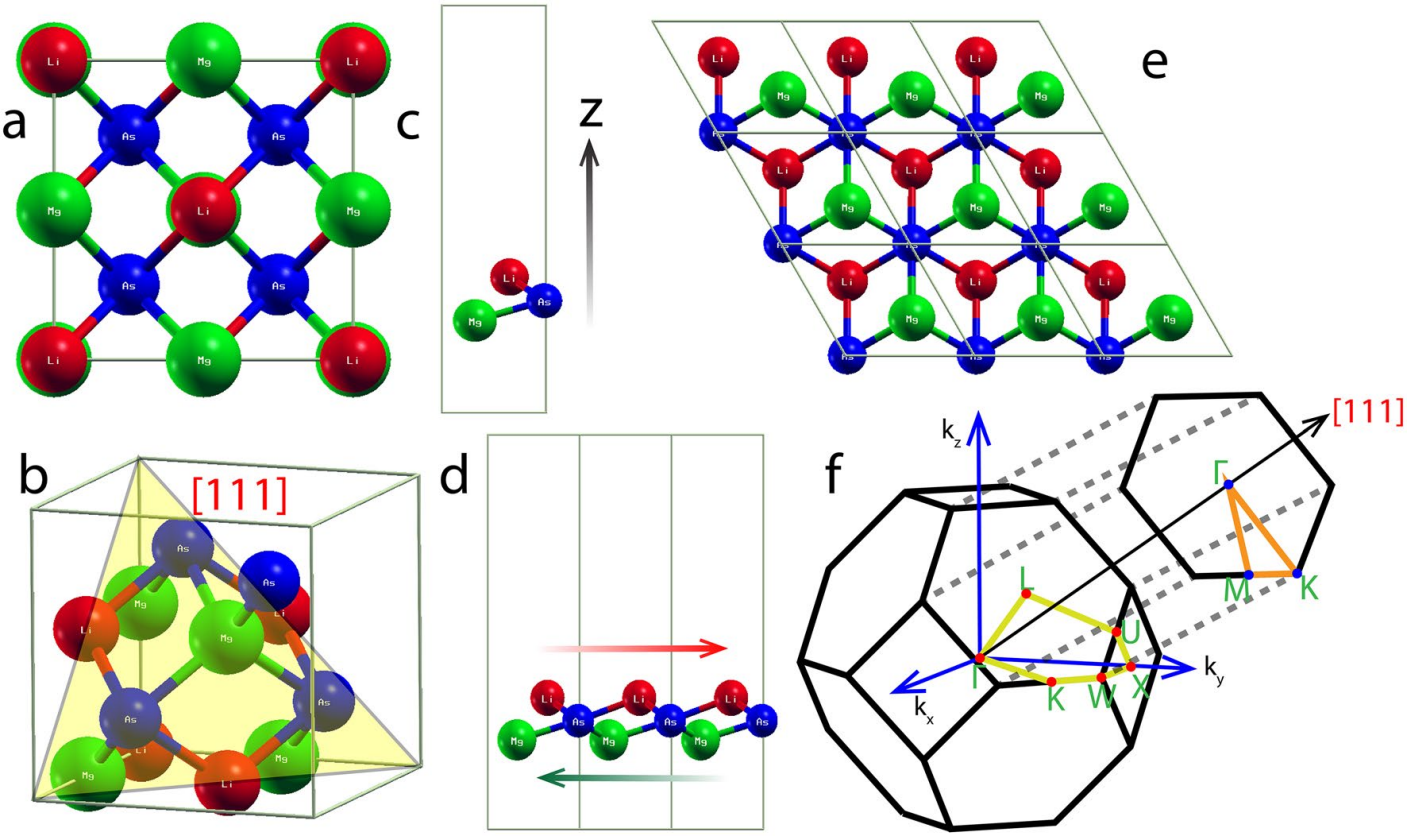
(**a**) Face Centered Cubic (FCC) structure of LiMgAs with; Li, Mg and As placed at, 4*b*, 4*c* and 4*a* Wyckoff positions respectively. (**b**) [111] plane of FCC LiMgAs. (**c**) Quantum confinement achieved by introducing 15 (Å) vacuum along z-direction of the [111] layer of LiMgAs which has structure similar to that of ${\text {1T-MoS}}_2$. (**d** and **e**) Side and Top views of the low-dimensional supercell. Figure (**d**) indicates the sandwich type structure with, Li atoms placed on top layer shifted towards right (red arrow) and Mg atom place in the bottom layer shifted towards left (green arrow) while the As atoms occupy the middle layer. (**f**) Irreducible first Brillouin Zones with the path for Electronic Band Structure indicated for the bulk and the low-dimensional [111] phase of LiMgAs.

In its bulk phase, LiMgAs has a FCC structure (Fig. 1a) with optimized lattice constant (a)=6.19 (Å), which matches exactly with the experimental value and other theoretical studies^21^. The structure is presented in Fig 1a with primitive cell vectors defined as ${\text {v}}_1 = (\text {a}/2)$(− 1,0,1), ${\text {v}}_2 = (\text {a}/2)$(0,1,1), ${\text {v}}_3 = (\text {a}/2)$(− 1,1,0). Here, the 4*b*, 4*c* and 4*a* Wyckoff positions^19^ are occupied by Li, Mg and As respectively with the interatomic distances for As-Mg and Mg-Li as 2.69 (Å) and 3.12 (Å) respectively.

For the very first time, we propose a novel low dimensional phase of HH LiMgAs by cleaving [111] crystal plane from the bulk FCC structure (Fig. 1b). This layer has trigonal structure similar to ${\text {1T-MoS}}_2$^39^ with optimized lattice constant 4.28 (Å) (Fig. 1c). The atomic arrangement is of sandwich type similar to ${\text {1T-MoS}}_2$. Here, the Li and Mg atoms occupy the top and bottom layers respectively while the As atoms are placed in the middle layer as shown in Fig. 1d,e. The interatomic distance of As-Mg is 2.58 (Å) and As-Li is 2.62 (Å) with sandwich height of 1.60 (Å). We achieve quantum confinement by introducing a vacuum of 15 (Å) along the z-direction to avoid interlayer interactions as shown in Fig. 1c.

**Figure 2:**
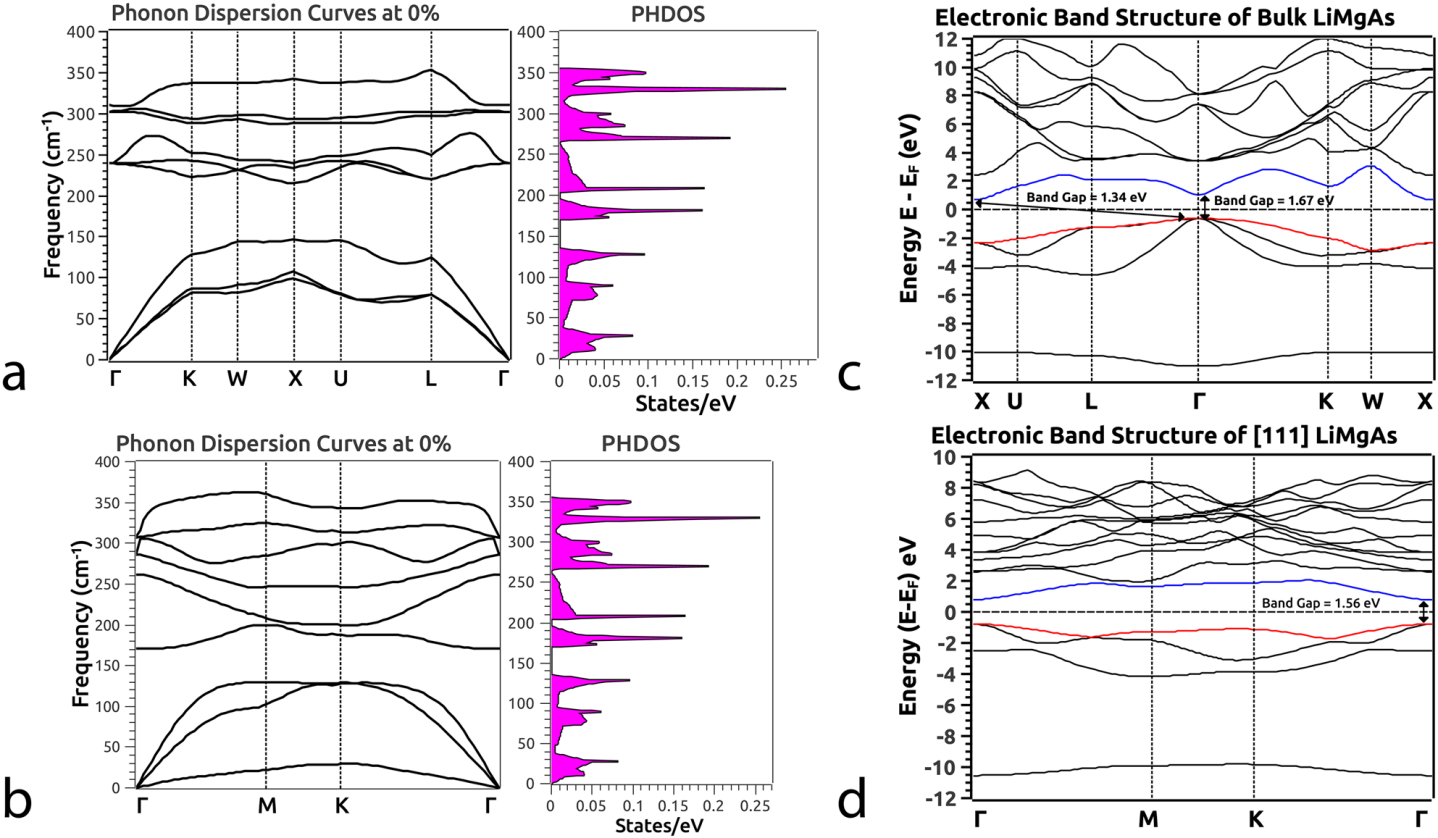
Phonon Dispersion Curves (PDC) at 0% pressure alongside the Phonon Density of States (PHDOS) for the (**a**) bulk and (**b**) low-dimensional [111] phase of LiMgAs. The absence of negative phonon frequencies in the entire Brillouin Zone indicates the systems are dynamically stable. Electronic Band Structure (EBS) of the (**c**) bulk and (**d**) low-dimensional [111] phase of LiMgAs.

Since the dynamic stability of proposed systems are quite important from a practical feasibility point of view, we performed Phonon calculations for both the phases of LiMgAs. Also, we calculated Phonon DOS (PHDOS) to ensure the absence of negative phonon frequencies in the entire BZ which indicates that the material is dynamically stable^44^. Since it is known that, the dynamical stability of a solid indicates increase in its potential energy against any combinations of the atomic displacements which is, equivalent to the condition that all phonons have real frequencies in harmonic approximation. However, when there is an imaginary frequency the system is said to be in dynamically unstable phase indicating decrease in potential energy close to the equilibrium atomic positions due to combination of atomic displacements^45–49^. Hence, these studies are important because, Phonon dynamics helps us unravel the governing vibrational dynamics of the system. The Phonon Dispersion Curves (PDC) and PHDOS are obtained as shown in Fig. 2 at 0% pressure for the (a) bulk and (b) low-dimensional [111] phase of LiMgAs. Since both the systems have an arrangement such that, three atoms occupy the unit cell, this gives rise to nine Phonon branches which is evident from the dispersion presented in Fig. 2a,b. It is evident from the PDC and PHDOS that, the bulk and the low-dimensional phase of HH LiMgAs are dynamically stable which implies they can be realized experimentally. In the bulk phase, the nine Phonon branches are comprised of, three in plane acoustic branches (one Longitudinal Acoustic (LA) and two Transverse Acoustic (TA) modes of vibration) dominant in the lower frequency regime and six optical branches (two Longitudinal Optical (LO) and four Transverse Optical (TO) modes of vibration) dominant in the higher frequency regime. Similarly, the low-dimensional phase exhibits nine phonon branches comprised of, three in plane acoustic branches, three optical branches and three out-of-plane acoustic (ZA) and optical (ZO) branches arising due to the vacuum (of 15 Å) provided along the z-direction.

**Figure 3:**
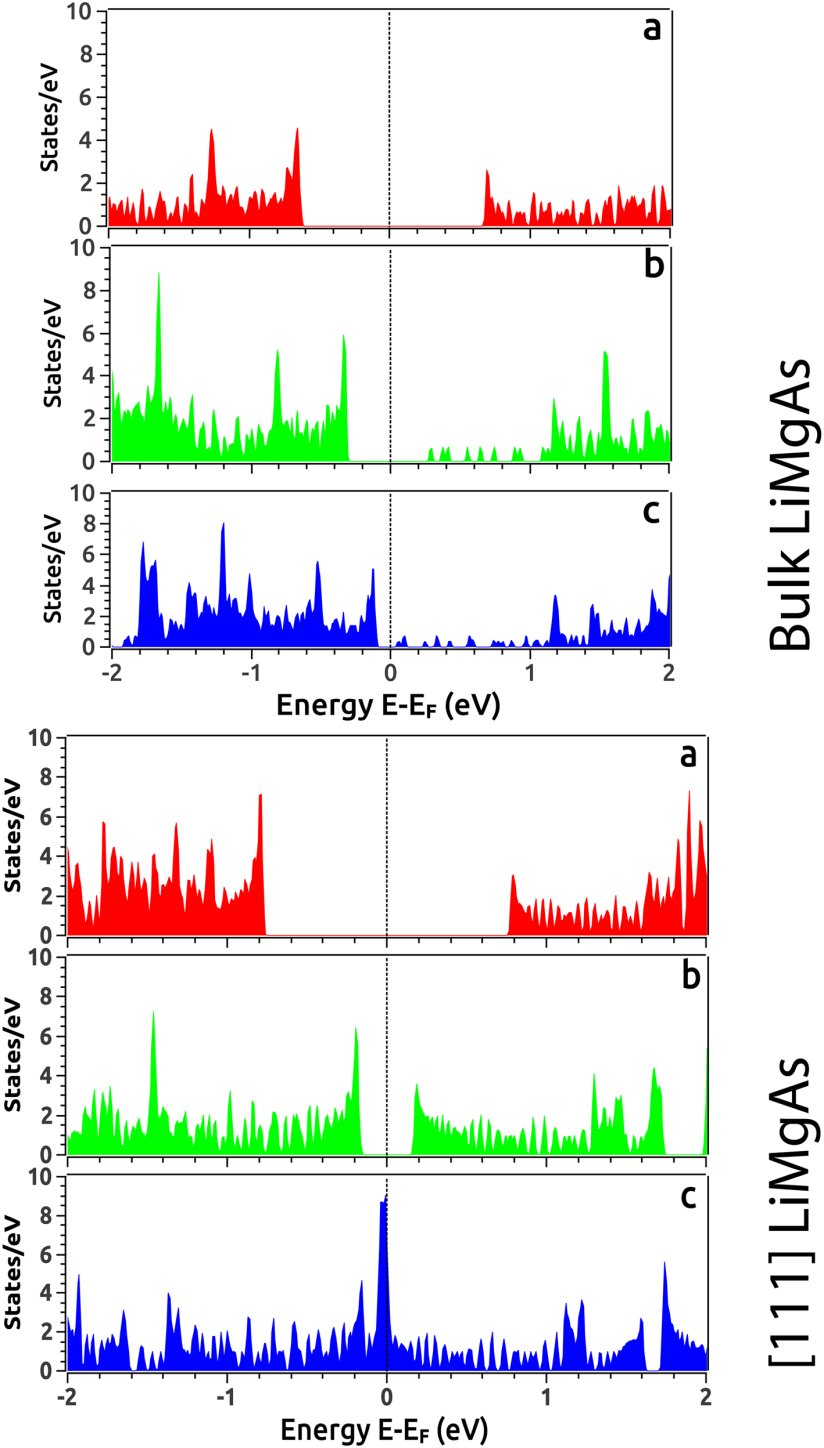
Density of States (DOS) for the bulk (top panel) and the low-dimensional [111] phase of LiMgAs (bottom panel) at (**a** − 0%, **b** − 8%, **c** − 15%) and (**a** − 0%, **b** − 8%, **c** − 10%) pressures respectively.

### Electronic properties

#### Bulk phase

**Figure 4:**
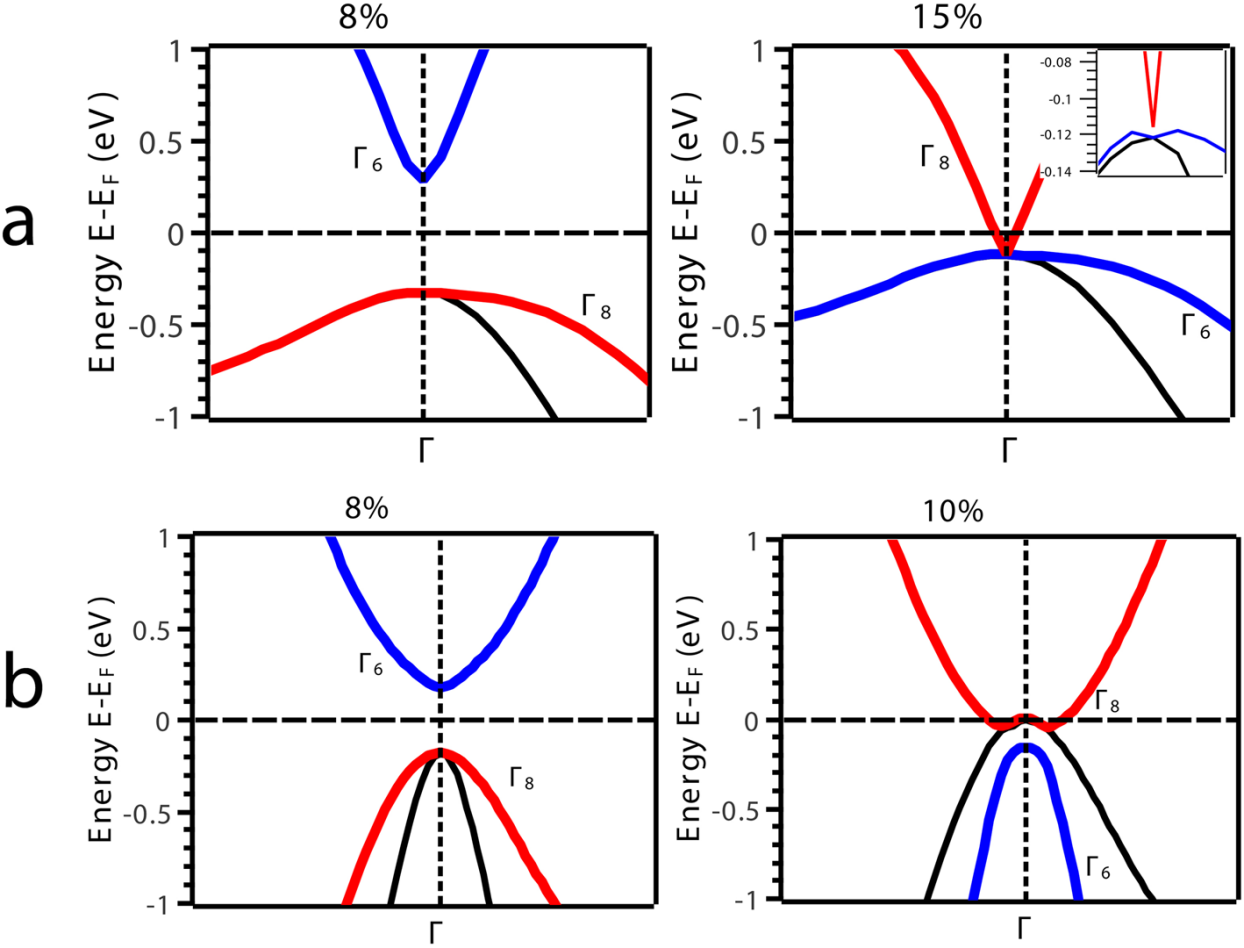
Electronic Band Structure (EBS) in the vicinity of Fermi level indicating the closing of band gap with band inversion in (**a**) bulk (inset, opening of the gap at 17% VEP) and (**b**) low-dimensional [111] phase of LiMgAs.

**Figure 5:**
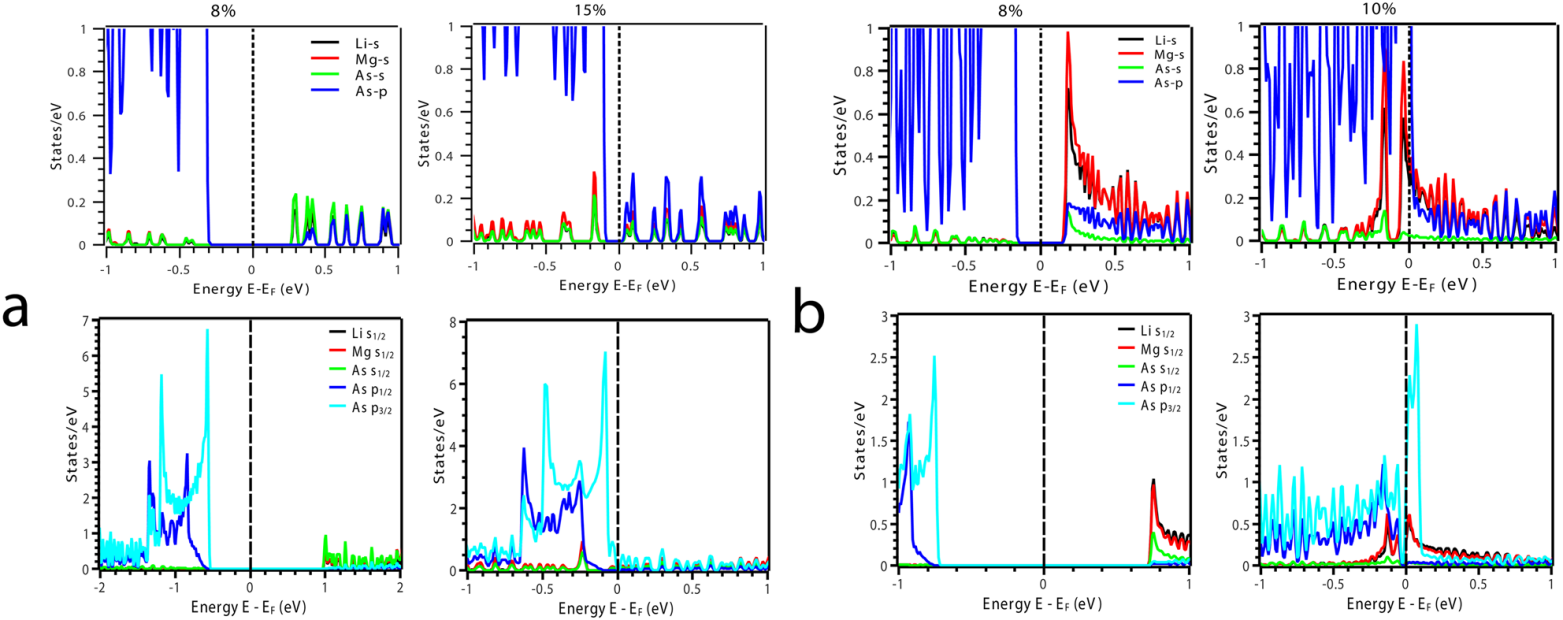
(**a**) Partial Density of States (PDOS) of the bulk phase of LiMgAs and (**b**) the low-dimensional [111] phase of LiMgAs (Top panel: without SOC, Bottom panel: with SOC) before and after critical pressure/strain indicating exchange of orbital contributions and orbital parity around Fermi.

The FCC structure in bulk phase exhibits indirect band gap in the entire BZ (Fig. 1f) evident from the electronic band dispersions as shown in Fig. 2c. For qualitative analysis of the TPT, we rely on the Electronic Band Structure (EBS) which is, a preliminary indicator of the band order inversion which is characteristic of the TI nature. At equilibrium, an indirect band gap of 1.34 eV exists along the BZ path X $\rightarrow$ U $\rightarrow$ L $\rightarrow$ $\Gamma$ and a direct band gap of 1.67 eV at $\Gamma$ (Fig. 2c) which is very close to 1.55 eV from previous theoretical work^21^. We could drive the system in two directions from here; (i) we either compress the system or (ii) expand it under VEP. We observed that with compression, the band gap continues to increase indicting perspective applications in the field of photovoltaic’s which is open for further exploration/investigations. Contrary to compression, the expansion driven by VEP unravels interesting properties. VEP mimics the isotropic pressure due to the presence of an intrinsic *void*, extreme internal *thermal* agitations and/or cavitation pressure arising from cavity nuclei^50^. The step increment in VEP leads to increase in the lattice volume eventually driving the system through a TPT i.e., the system undergoes phase transition from semi-conducting to semi-metallic as observed from Density of States (DOS) at 0%, 8% and 15% represented in Fig. 3. As VEP is increased, the band gap reduces and becomes direct along the high symmetry point $\Gamma$ in the BZ as shown in Fig. 4a. At a critical VEP of 15% which gives rise to a pressure of 8.18 GPa in the unit cell, a semi-metallic Dirac dispersion along $\Gamma$ in the BZ is observed (Fig. 4a) giving rise to surface states (Fig. 7a). Beyond 15% VEP at 17% (inset Fig. 4a), we observe reopening of the band gap indicating the exchange of orbital nature in the vicinity of Fermi level (${\text {E}}_F$). This mechanism of orbital exchange is clearly evident from the Projected Density of States (PDOS) as shown in Fig. 5a. Prior to the critical VEP, the Conduction Band (CB) is dominated by the ‘s’ orbital contributions from Li, Mg and As whereas the Valence Band (VB) is purely dominated by the contributions from the ‘p’ orbital of As. The ‘s’ orbitals of Li, Mg and As exhibit a strong hybridization with the ‘p’ orbital of As deeper into the CB as compared to the VB. With the formation of Dirac cone, a non-negligible increment in the ‘p’ and ‘s’ orbital contributions across the Fermi level is observed. The ‘p’ orbital contribution from As rises in CB retaining its strong hybridization with the ‘s’ orbitals of Li, Mg and As. Although, the ‘s’ orbital contributions from Li, Mg and As increase fairly in the VB but, the domination of ‘p’ orbital from As remains the same. Such inversion is quite weak because, the partial occupancy of Fermions arising from the ‘p’ orbital of As is evident in the conduction band minima indicating a linear Dirac dispersion (with velocity of Fermions ${\text {v}}_f = \partial {E}/\hslash \partial {k}$^51^, $\sim 0.06  \,^{*}\,10^{5} {\text {ms}}^{-1}$ and $\sim 0.64 \,^{*}\,10^{5} {\text {ms}}^{-1}$ along the L $\rightarrow$ $\Gamma$ and $\Gamma$ $\rightarrow$ K directions respectively in the BZ) but, the non-linear/parabolic dispersion of valence band maxima indicates that Fermion transport occurs at lower velocities (with ${\text {v}}_f$^51^ $\sim 0.04 \,^{*}\,10^{5} {\text {ms}}^{-1}$ and $\sim 0.33 \,^{*}\,10^{5} {\text {ms}}^{-1}$ along the L $\rightarrow$ $\Gamma$ and $\Gamma$ $\rightarrow$ K directions respectively in the BZ). We estimate the band inversion strength ($\Delta$)^36,37^, for the bulk case, which is quite weak (i.e., $\Delta$ $\sim$ 7.0 meV) but, since As is a heavy element the SOC will have prominent effects on the band gap. Here, we find that, the SOC opens a band gap of $\sim$ 1.5 meV at the Dirac point along $\Gamma$ in the BZ at 15% VEP which is quite small. The PDOS with SOC at 8% and 15% VEP (Fig. 5a) indicates pronounced exchange of orbital contributions (parity of orbitals included) around the Fermi level (E_*F*_).

#### Low dimensional phase

As compared to the bulk phase, the low-dimensional phase of LiMgAs is intrinsically semi-conducting with a direct band gap of 1.56 eV as seen from Fig. 2d. This feature is quite different as compared to the bulk parent which has slightly smaller *indirect* band gap. We can attribute this to the quantum size effect due to the one dimensional confinement of the system. We observe degeneracies in the valence band maxima’s along the high symmetry paths, L $\rightarrow$ $\Gamma$ and W $\rightarrow$ X while non-degenerate along the paths, X $\rightarrow$ U $\rightarrow$ L and $\Gamma$ $\rightarrow$ K $\rightarrow$ W in the vicinity of Fermi level (Fig. 2d). This feature is exactly similar to the bulk parent. The valence band maxima and the conduction band minima are completely non-degenerate throughout the BZ in the low-dimensional as well as bulk phase (Fig. 2c–d). When we subject the monolayer to isotropic compressive pressure we observe that, the band gap continues to reduce forming a Dirac cone like dispersion between 8% to 10% compressive pressure along the high symmetry point $\Gamma$ in the BZ as seen in Fig. 4b. The Dirac dispersion of conduction band minima exhibits Fermion velocities, ${\text {v}}_f$^51^ $\sim 0.56 \,^{*}\,10^{5} {\text {ms}}^{-1}$ and $\sim 11.13 \,^{*}\,10^{5} {\text {ms}}^{-1}$ along K $\rightarrow$ $\Gamma$ and $\Gamma$ $\rightarrow$ M direction respectively in the BZ. While, the valence band maxima exhibits Fermion velocities, ${\text {v}}_f$^51^ $\sim 1.31 \,^{*}\,10^{5} {\text {ms}}^{-1}$ and $\sim 7.64 \,^{*}\,10^{5} {\text {ms}}^{-1}$ along K $\rightarrow$ $\Gamma$ and $\Gamma$ $\rightarrow$ M direction respectively in the BZ. This implies that, the transport of Fermions is facilitated at higher velocities as compared to the bulk phase. The Dirac dispersion along $\Gamma$ indicates towards the conduction along the edge states while the bulk remains insulating throughout the BZ. Also we can observe that, the degeneracy of bands in the valence band maxima at $\Gamma$ lifts off contributing Fermions in the vacant conduction band. With further increment in the compressive pressure we observe TPT i.e., the Dirac degeneracy at $\Gamma$ lifts off reopening the band indicating TI nature. The critical pressure (10%) at which we observe the TPT in low-dimensional phase is 2.96 GPa which is quite small as compared to the bulk parent. The phase transition from semi-conducting to semi-metallic can also be observed from the DOS at 0%, 8% and 10% represented in Fig. 3. We analyze the PDOS to address the band inversion mechanism in terms of the orbital contributions of Li, Mg and As in the vicinity of Fermi level (${\text {E}}_F$). From the PDOS shown in Fig. 5b, we can estimate that, the band inversion is quite strong as compared to that in the bulk phase.

**Figure 6:**
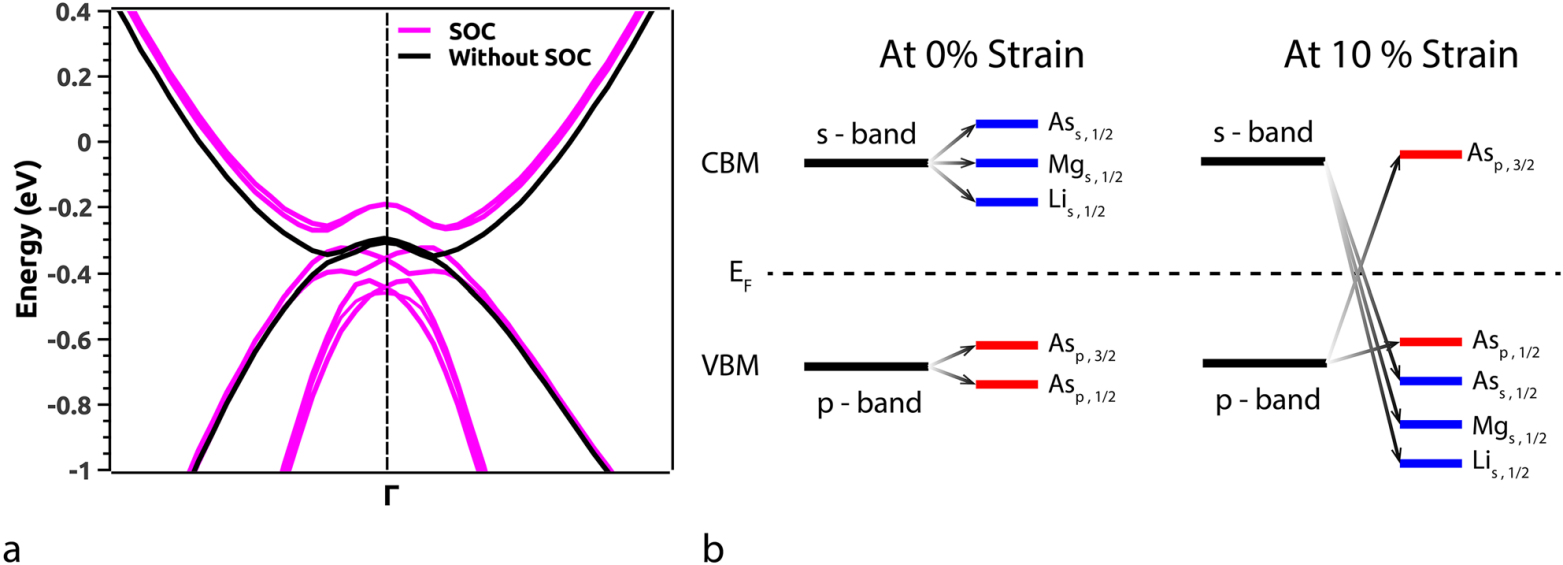
(**a**) Electronic Band Structure (EBS) of [111] LiMgAs without and with SOC indicating a strong band inversion at 10% compressive strain. (**b**) Orbital mechanism of band inversion in [111] LiMgAs due to SOC at 0% and 10% strain.

The low-dimensional system exhibits a typical ‘p’ orbital dominated VB and ‘s’ orbital dominated CB. At the critical compressive pressure, the ‘s’ orbitals from CB penetrate the VB while, the ‘p’ orbital contribution from VB dominates the states in CB. Such band order is similar to that of HgTe under adiabatic continuation approach^36^. This indicates that, the Fermions arising from the ‘p’ orbital of As are major contributors to the observed TI nature. Since, the density of Fermions is quite high at the Fermi level (E_*F*_), we can expect a non-trivial topological nature in this low dimensional phase as compared to the bulk phase. Also, the band inversion strength^36,37^ for the low dimensional phase is positive and strong i.e., $\Delta$ $\sim$ 113.7 meV as compared to the bulk parent which indicates that, the low dimensional phase can be a TI. Also, the SOC effects are more pronounced in this phase due to buckling^33^ (evident from the PDOS with SOC as shown in Fig. 5b) as compared to the bulk parent. The band gap due to SOC is $\sim$ 55.0 meV which is superior to graphene, silicene and arsenene^34,52^. Figure 6a represents the band inversion due to SOC alongside (Fig. 6b) the inversion mechanism in terms of orbital contributions at 0% and 10% pressure. This band gap indicates that the proposed low dimensional phase can be used as a spintronic material, since it is known that, topological insulators exhibit spin polarization^43^ which can be enhanced and fine tuned by strain engineering for spintronic applications^53^. Such applications are proposed under strained conditions because, the material undergoes TPT at a particular strain/pressure.

### ${\mathbb {Z}}_2$ analysis, surface and edge states

**Figure 7:**
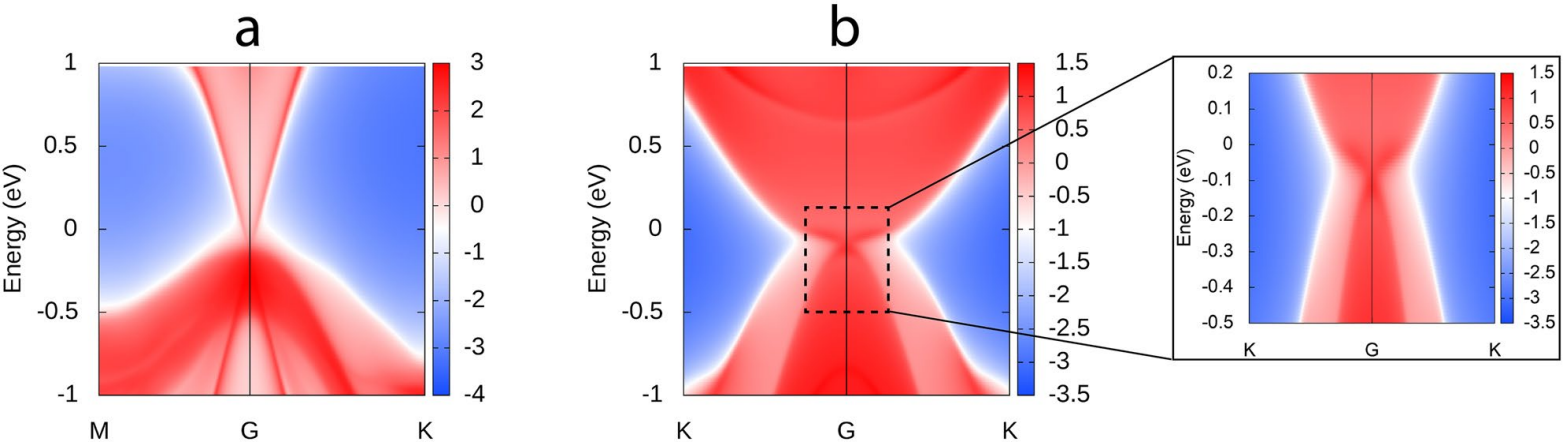
Computational surface and edge states of the (**a**) Bulk and (**b**) Low dimensional [111] phase of HH LiMgAs respectively (alongside zoomed image of the edge states in a small energy range).

Figure 7a,b represents, the surface and edge states at the critical pressure which is obtained computationally using, iterative Green’s function calculation for the bulk and low-dimensional phase of LiMgAs. This result compliments our electronic analysis, indicating a single surface state along the high symmetry point $\Gamma$ in the BZ. We quantify our results by calculating the ${\mathbb {Z}}_2$ invariant to identify the topological strength and class. By virtue of the FCC structure, the bulk phase of LiMgAs lacks inversion symmetry hence, we calculate the ${\mathbb {Z}}_2$ invariant along two momentum planes in the BZ using the formula presented in equation 1 and 2. For this purpose, we utilize the Maximally Localised Wannier Functions (MLWF) to create a Tight Binding (TB) model. Using this model, the ${\mathbb {Z}}_2$ is calculated around the Wannier Charge Centres (WCC) in the vicinity of Fermi level (E_*F*_). The ${\mathbb {Z}}_2$ indices ($\nu _0$, $\nu _1$ $\nu _2$ $\nu _3$) were found to be $\big (0, 0 0 0\big )$ which indicates that, the bulk phase of LiMgAs is trivial insulator (with a possibility of non-trivial nature in a low dimensional form).

1 $$\begin{aligned} \nu _0& = {} \Big [ \big ( {\mathbb {Z}}_2 \big )_{(k_i=0)} + \big ( {\mathbb {Z}}_2 \big )_{(k_i=0.5)} \Big ] \big ( mod 2 \big ) \end{aligned}$$

2 $$\begin{aligned} \nu _i& = {} \big ({\mathbb {Z}}_2 \big )_{(k_i=0.5)} \end{aligned}$$

Following the calculation of edge states at the critical compressive pressure (Fig. 7b) for the low-dimensional phase, we quantified our electronic analysis by calculating the ${\mathbb {Z}}_2$ invariant ‘$\nu$’ to identify the topological strength. Since the system has a hexagonal structure, it is governed by inversion symmetry. This implies that, we can calculate the ${\mathbb {Z}}_2$ invariant by taking the product of parities of occupied eigen states at four Time Reversal Invariant Momentum (TRIM) ($K_s$) points which was proposed by Fu et al.^2^ i.e., $\nu$ is obtained using the formula presented in equations 3 and 4 where, $\zeta^{s} _{p}$ are the parity of eigen functions for ‘N’ occupied bands (which are not doubly degenerate since, SOC is not considered) and $\delta (K_s)$ are the product of parities of eigen values at TRIM points. The ${\mathbb {Z}}_2$ invariant ‘$\nu$’ can also be calculated around WCC (in the vicinity of Fermi level) using the TB model derived from the MLWF. We find that, the system is a TI as compared to the bulk phase and is characterized by ${\mathbb {Z}}_2$ invariant $\nu$ = 1.

3 $$\begin{aligned} \delta (K_s)& = {} \prod _{p=1}^N \zeta _p^s \end{aligned}$$

4 $$\begin{aligned} (- 1)^{\nu }& = {} \prod _{s=1}^4 \delta (K_s) \end{aligned}$$

With this, we conclude from our Density Functional Theory based *first-principles* calculations that, a TI can be realized by dimensional confinement of a crystal plane from a bulk material which exhibits trivial nature. The HH complex LiMgAs under study here is, dynamically stable with a FCC structure. We find that, this structure exhibits a trivial nature when it undergoes a TPT driven by VEP. This implies that, it cannot effectively be used as a TI applications at room temperature since the surface states wont be robust against external perturbations. As compared to this, we predict a novel low-dimensional and dynamically stable form of LiMgAs which turns out to be a TI under compressive pressure. Both, the bulk and low-dimensional phases exhibit odd number of band inversions in BZ due to the exchange of orbital features between VB and CB which was studied electronically from EBS, DOS and PDOS. Under VEP the bulk phase undergoes phase transition from, semi-conducting to semi-metallic whereas; the low-dimensional phase undergoes a phase transition from, semi-conducting to semi-metallic under the influence of compressive pressure. The drastic difference in the properties of the two phases arises due to dimensionality of the system and the dominance of quantum confinement effects in the low-dimensional phase. The ${\mathbb {Z}}_2$ invariant for bulk and low-dimensional phase are, $\big (\nu _0, \nu _1 \, \nu _2 \, \nu _3 \big ) \equiv \big (0, 0 0 0\big )$ and $\nu$ = 1 respectively. With our calculations, we pave a new method of dimensional engineering to achieve TI nature in materials with perspective applications in spintronics and nano-electronics.

## Methods

We perform electronic studies using, Density Functional Theory based *first-principles* calculations in QUANTUM ESPRESSO code^54^ which implements Plane Wave Self Consistent Formulation (PWSCF). We employed norm conserving generalized gradient approximation (GGA) pseudopotentials which utilize the, ${\text {1s}}^1$, ${\text {3s}}^2$ and ${\text {4s}}^2 {\text {4p}}^3$ orbital contributions of Li, Mg and As respectively. Martins-Troullier pseudopotential method is used with exchange correlation of Perdew-Burke-Ernzerhof (PBE) functional type^55^. Projector augmented wave (PAW)^56^ sets with PBE exchange correlation^55^ are used for SOC calculations since, it considers the relativistic effects arising from the core electrons. Lattice constant, wave function kinetic energy cut-off and k-mesh were optimized by performing convergence test with, the test condition of total pressure on atoms as 0.00 kbar. The wave function kinetic energy cut-off was obtained to be 90 Ry (90 Ry for PAW) and 50 Ry (80 Ry for PAW) for the bulk and low dimensional structures respectively. Uniform momentum grid of Monkhorst-Pack Grid type^57^ was used in the calculations with, 8 $\times$ 8 $\times$ 8 (8 $\times$ 8 $\times$ 8 for PAW) and 6 $\times$ 6 $\times$ 1 (8 $\times$ 8 $\times$ 1 for PAW) for bulk and low dimensional structures respectively. Structural stability was verified by calculating PDC using Density Functional Perturbation Theory^58^. A q-mesh for bulk and low dimensional structures of 6 $\times$ 6 $\times$ 6 and 5 $\times$ 5 $\times$ 1 were used respectively. With thorough qualitative analysis, we quantify our results by calculating the ${\mathbb {Z}}_2$ invariant using Wannier90 (W90)^59^, around the Wilson loops. This is followed by the plotting computational ARPES like surface states using WannierTools (WT)^60^ which indicates surface/edge states.
