# Author Correction: Dimensional engineering of a topological insulating phase in Half-Heusler LiMgAs

**DOI:** 10.1038/s41598-021-95885-9

**Published:** 2021-08-12

**Authors:** Raghottam M. Sattigeri, Prafulla K. Jha

**Affiliations:** Department of Physics, Faculty of Science, The Maharaja Sayajirao University of Baroda, Vadodara, Gujarat 390002 India

Correction to: *Scientific Reports* https://doi.org/10.1038/s41598-021-85806-1, published online 19 March 2021

The original version of this Article contained a repeated error, where a factor of 10^5^ for the v_*f*_ values of Fermions was omitted.

As a result, in the Results and discussion section, under the subheading “Electronic properties”, “Bulk phase”,

“Such inversion is quite weak because, the partial occupancy of Fermions arising from the ‘p’ orbital of As is evident in the conduction band minima indicating a linear Dirac dispersion (with velocity of Fermions v_*f*_ = ∂E/ℏ∂k^51^, ~ 0.06 ms^−1^ and ~ 0.64 ms^−1^ along the L → Γ and Γ → K directions respectively in the BZ) but, the non-linear/parabolic dispersion of valence band maxima indicates that Fermion transport occurs at lower velocities (with v_*f*_^51^ ~ 0.04 ms^−1^ and ~ 0.33 ms^−1^ along the L → Γ and Γ → K directions respectively in the BZ).”

now reads:

“Such inversion is quite weak because, the partial occupancy of Fermions arising from the ‘p’ orbital of As is evident in the conduction band minima indicating a linear Dirac dispersion (with velocity of Fermions v_*f*_ = ∂E/ℏ∂k^51^, ~ 0.06 * 10^5^ ms^−1^ and ~ 0.64 * 10^5^ ms^−1^ along the L → Γ and Γ → K directions respectively in the BZ) but, the non-linear/parabolic dispersion of valence band maxima indicates that Fermion transport occurs at lower velocities (with v_*f*_^51^ ~ 0.04 * 10^5^ ms^−1^ and ~ 0.33 * 10^5^ ms^−1^ along the L → Γ and Γ → K directions respectively in the BZ).”

Under the same section, under the subheading “Low dimensional phase”,

“The Dirac dispersion of conduction band minima exhibits Fermion velocities, v_*f*_^51^ ~ 0.56 ms^−1^ and ~ 1.31 ms^−1^ along K → Γ and Γ → M direction respectively in the BZ. While, the valence band maxima exhibits Fermion velocities, v_*f*_^51^ ~ 11.13 ms^−1^ and ~ 7.64 ms^−1^ along K → Γ and Γ → M direction respectively in the BZ.”

now reads:

“The Dirac dispersion of conduction band minima exhibits Fermion velocities, v_*f*_^51^ ~ 0.56 * 10^5^ ms^−1^ and ~ 11.31 * 10^5^ ms^−1^ along K → Γ and Γ → M direction respectively in the BZ. While, the valence band maxima exhibits Fermion velocities, v_*f*_^51^ ~ 1.31 * 10^5^ ms^−1^ and ~ 7.64 * 10^5^ ms^−1^ along K → Γ and Γ → M direction respectively in the BZ.”

The original Article has been corrected.

